# Optimization of the Care4Today Digital Health Platform to Enhance Self-Reporting of Medication Adherence and Health Experiences in Patients With Coronary or Peripheral Artery Disease: Mixed Methods Study

**DOI:** 10.2196/56053

**Published:** 2025-03-17

**Authors:** Stephanie Juan, Ante Harxhi, Simrati Kaul, Breeana Woods, Monica Tran, Gabrielle Geonnotti, Archit Gupta, Emily Dean, Cassandra E Saunders, Gloria Payne

**Affiliations:** 1 Janssen Scientific Affairs, LLC Titusville, NJ United States; 2 Johnson & Johnson Technology Services Titusville, NJ United States; 3 ZS Associates San Francisco, CA United States; 4 CorEvitas, part of ThermoFisher Scientific Waltham, MA United States; 5 Participant in Janssen Patient Engagement Research Council Philadelphia, PA United States; 6 Participant in Janssen Patient Engagement Research Council Maynard, MA United States

**Keywords:** app, cardiovascular disease, Care4Today, coronary artery disease, digital health, health tracker, medication reminder, mobile health, mHealth, qualitative, peripheral artery disease

## Abstract

**Background:**

Care4Today is a digital health platform developed by Johnson & Johnson comprising a patient mobile app (Care4Today Connect), a health care provider (HCP) portal, and an educational website. It aims to improve medication adherence; enable self-reporting of health experiences; provide patient education; enhance connection with HCPs; and facilitate data and analytics learning across disease areas, including cardiovascular disease.

**Objective:**

This study aimed to gather patient feedback on Care4Today Connect, specifically the coronary artery disease (CAD) and peripheral artery disease (PAD) module, and to cocreate and validate features with patients to optimize the app experience for those with CAD, PAD, or both.

**Methods:**

We conducted 3 research engagements between November 2022 and May 2023. Participants were US-based adults recruited and consented through the sponsor’s Patient Engagement Research Council program. Participants self-reported a diagnosis of cardiovascular disease, and in some cases, specifically, CAD, PAD, or both. Part 1, internet survey, posed quantitative questions with Likert-scale answer options about existing app features. Part 2, virtual focus group, and part 3, virtual individual interviews, both used semistructured qualitative discussion to cocreate and validate new app enhancements. The quantitative data from part 1 was evaluated descriptively to categorize mobile health app use, confidence in the ability to use the app, and motivations for app use. The qualitative discussions from parts 2 and 3 were synthesized to understand participants’ app needs and preferences to inform an optimal app experience.

**Results:**

The response rate for part 1, internet survey, was 67% (37/55). Most participants felt at least somewhat confident using the app after seeing the newly added app tutorial (33/37, 89%), and at least somewhat confident in their ability to earn points for completing activities using app instructions (33/37, 89%). In part 2, virtual focus group (n=3), and part 3, virtual individual interviews (n=8), participants collectively preferred to enhance the app with (1) the ability to automatically add medication data for tracking and (2) the ability to receive relevant care team feedback on their self-reported health experiences. Participants would be willing to spend 10-15 minutes a day tracking 4-5 health experiences, especially those requested by their HCP.

**Conclusions:**

Participants prefer apps that can reduce user burden and provide information relevant to them. Care4Today Connect can optimize the user experience for patients with CAD, PAD, or both with the automatic addition of medication data for tracking and in-app care team feedback on patient self-reported health experiences.

## Introduction

### Overview

With the widespread use of mobile health (mHealth) apps and wearable fitness trackers, many people routinely self-report personal health experiences (eg, physical activity, sleep, and mood). In the health care context, self-reported data are useful for shared decision-making, providing clinicians with a more holistic perspective of patient health beyond office visits and hospitalizations, improving communication, enhancing coordination of care, and increasing patient engagement [1]. Digital health technology has the potential to become an important part of health care systems, promoting behavior change, enhancing medication adherence, and improving health outcomes in chronic conditions such as cardiovascular disease [2].

More than 18 million adults in the United States have coronary artery disease (CAD) [3], and up to 42% of these people also have peripheral artery disease (PAD) [4,5]. CAD remains the leading cause of death in the United States, accounting for 1 in every 4 deaths [6], and medication nonadherence is linked to poor outcomes [7]. Patients with CAD, PAD, or both often take multiple medications to control their disease and other comorbid conditions. The prevalence of polypharmacy (typically defined as simultaneous use of ≥5 medications [8]) is estimated to be 17% among US adults, 40% to 62% in those with heart disease [9], and 91% in patients with CAD [10]. Polypharmacy has been linked to both medication errors [11] and nonadherence [12].

In CAD, mHealth apps have been shown to support secondary prevention lifestyle changes [13], with positive effects on medication adherence, exercise and physical activity, quality of life, major adverse cardiovascular outcomes, and hospital readmissions [14-16]. In PAD, mHealth technologies have been used successfully to improve health behavior, providing motivation to exercise through activity monitoring and coaching, and have been linked to changes in both health outcomes and disease coping [17].

### Care4Today

Care4Today is a digital health platform initially launched by Johnson & Johnson as a medication reminder app in 2013. Today, the platform has expanded to 3 components: a patient mobile app, a health care provider (HCP) portal, and an educational website. The app (Care4Today Connect [18]) has been designed to encourage patients to take an active role in managing their overall health. According to the sponsor’s internal health store database and Google Analytics, from mid-2020 until mid-2024, the app has supported an estimated 2000 users across company-sponsored initiatives. Features include medication and appointment reminders; various self-reported health experience trackers, including elective biometrics, health, and lifestyle activity with visual trends over time; and educational resources tailored toward specific disease management. Users can access scheduled health activities and resources related to their disease and can share data on their progress with their care team. Access to the app is granted to users in the United States with a code provided by their HCP across multiple disease areas, including cardiovascular disease [18]. It is available for both iOS and Android users; is available in English and Spanish; and can connect to fitness apps like HealthKit, Google Fit, and Fitbit but does not require a wearable device.

The Care4Today HCP portal allows the care team to view patient self-reported health experiences shared through the mobile app. The portal enables the care team to assign, monitor, and adjust patient care (eg, medications, appointments, education, and trackers) in real time, as well as to send in-app reminders and encouragement to their patients. The Care4Today website [18] provides additional educational resources accessible to both patients and HCPs. A cardiovascular health-specific webpage was created to complement the CAD- and PAD-specific care modules for the app.

Patient cocreation and validation are essential for optimizing the mobile app experience and app usefulness for managing disease. Quantitative surveys are a valuable means of capturing patient feedback, while qualitative studies can provide rich context about patient perspectives, the user experience, and barriers to using apps for health management. We conducted a 3-part, exploratory study to optimize the Care4Today Connect app and digital health platform for people living with CAD, PAD, or both, via a mixed methods approach involving both quantitative and qualitative components.

## Methods

### Ethical Considerations

A consent and release form was signed by the participants that communicated confidentiality and Health Insurance Portability and Accountability Act (HIPAA)–compliant practices. This study (institutional review board [IRB] ID 12459-EDean) was assessed by Sterling IRB (Atlanta, GA) and determined to be exempt from IRB review (45 C.F.R. §46.104(d)) under the Department of Health and Human Services category 2 exemption. The purpose of this study was to collect personal perspectives and qualitative insights from the participants. The study was also conducted in accordance with the Helsinki Declaration of 1964 and its later amendments. The study was voluntary, and all participants were compensated for their time.

### Study Design

This exploratory sequential research was conducted in three parts: part 1, internet survey, to gain patient feedback on existing features of the Care4Today Connect app; part 2, virtual focus group, in which participants collectively helped to cocreate and envision app enhancements; and part 3, virtual individual interviews, to validate prototype app enhancements discussed in part 2.

### Participant Recruitment

Adults with cardiovascular disease residing in the United States were recruited and consented through the sponsor’s Patient Engagement Research Council (PERC) program. PERCs constitute groups of disease-aware individuals living with chronic health conditions in the United States [19,20]. People with a range of health care experiences are recruited based on clinical, demographic, and epidemiologic criteria through various channels, including outreach to patient advocacy organizations, digital advertisements, social media, and physician referrals. PERC members come together to share their experiences and insights of a common diagnosis through a structured series of specific engagement activities.

Eligible participants for all 3 parts of this study were members of the sponsor’s PERC who self-reported having a diagnosed cardiovascular condition. Purposeful sampling was used to ensure racial and ethnic diversity across all parts of the study. Full eligibility criteria for PERC members are described in Multimedia Appendix 1. In part 2, purposeful sampling was used to ensure that all participants were taking >1 medication (self-reported) and that a variety of experience levels with mHealth apps was represented.

### Procedures

#### Part 1: Internet Survey

Part 1, internet survey, was conducted with participants with cardiovascular disease, including those with CAD, PAD, or both, between November 28 and December 2, 2022. Eligible participants were invited to participate via email and received a survey link programmed using Alchemer software. CorEvitas designed the survey to be completed within 25 minutes. The aim was to assess respondent’s understanding of how to use existing app features. It consisted of 33 questions across 5 categories, including Upfront, Tutorial for New Users, Earned Points, App in Clinical Study, and Overall. Three “Upfront” questions focused on the demographics and clinical characteristics of respondents, and their experience with mHealth apps. The “Tutorial for New Users” category included 18 questions asking the respondent to review tutorial screenshots of how to navigate the app as well as indicate their understanding of each component. The “Earned Points” category included 4 questions asking the respondent to review app screenshots on how to earn points for completing app activities and indicate their understanding of each component. They were also asked to share their opinions on the concept of earning points for completing activities in the app. The “App in Clinical Study” included 6 questions about motivations for taking part in a clinical study using mHealth apps (Multimedia Appendix 2). The “Overall” category included 2 questions asking the respondent to indicate how likely they would be to recommend the app to a friend or coworker. The rating scale was 1 to 10, where 1 was unlikely and 10 was very likely. For most questions, multiselect or 5-point, Likert-scale response options of agree to disagree, or not at all confident, to very confident were provided, including an option to choose “Other” and elaborate in a free-text response.

#### Part 2: Cocreation

Part 2, virtual focus group, was held on April 13, 2023, with participants with CAD, PAD, or both. The aim was to cocreate concepts with a small group of participants. Design and facilitation were led jointly by researchers from CorEvitas and ZS Associates. During the 2-hour session, participants were given an overview of the Care4Today Connect app and were asked to discuss features that may enhance the user experience. A semistructured discussion guide focused the session on two initiatives: (1) features that could improve how medication data are added to the app to ensure correct prescribed medications are tracked, alleviate user burden of manual input, and reduce input error; and (2) features for improved sharing of self-reported health experiences that could be used to facilitate feedback from care teams. To aid discussions, additional information was shared with the group, including screenshots of the existing feature for adding medication data (Multimedia Appendix 3) and illustrative mock-ups of how new medication, as well as health experience tracking features that might be incorporated into the app (Multimedia Appendix 4). For adding medication data, 2 options were presented; option 1 leveraged third-party insurance portal while option 2 used optical character recognition (OCR) technology, which involves the user taking an image of a medication bottle and then converting that image to readable text [21]. For self-reporting of health experiences, the existing method for tracking this data was presented.

#### Part 3: Validation

Part 3 of the research aimed to validate the enhancements cocreated with patients during the virtual focus group in part 2. One-hour virtual interviews were conducted between May 2 and 4, 2023, with participants with CAD, PAD, or both. Design and facilitation were led jointly by researchers from CorEvitas and ZS Associates. Discussions were structured around two enhancements identified in part 2: (1) auto-add medication data via the insurance portal and OCR; and (2) a “For You” tab with notifications, and personalized feedback about trends in their self-reported medication or health experiences tracking. To help with this, visuals were provided of Care4Today Connect app prototypes (Multimedia Appendix 5), and a semistructured discussion guide (Multimedia Appendix 6) was used to focus the agenda. Participants were asked to rate the perceived value of, and their willingness to use, the proposed features on a 7-point Likert scale (1=not at all likely; 7=highly likely).

### Data Collection

All participants provided insight into their current experience with medication and health experience tracking and their prior use of mHealth apps. Demographic information was also collected in the part 1 web-based survey. All sessions were audio recorded and transcribed.

### Analysis

#### Part 1: Internet Survey

Quantitative analysis was applied to summarize collective responses in Microsoft Excel. The goal of the analysis was to assess the user’s understanding of how to use existing app features. A senior patient experience research specialist from CorEvitas reviewed and presented the data descriptively as frequency and percentage.

#### Part 2: Focus Group and Part 3: Individual Interviews

Qualitative analysis identified patient insights and preferences directly applicable to the Care4Today app. The goal of the analysis was to detail the recommended features to be incorporated into a future version of the app. The team of senior research specialists and product designers from ZS Associates directly observed and analyzed the data. Patient insights were synthesized by using a directed content approach where inputs were systematically mapped to potential app functionalities presented during each session. The data were then further categorized by user appeal, task ease, and privacy concerns, and then finally synthesized to inform whether to enhance, modify, or deprioritize discussed C4T enhancements. No formal coding was used.

## Results

### Overview

**Figure 1:**
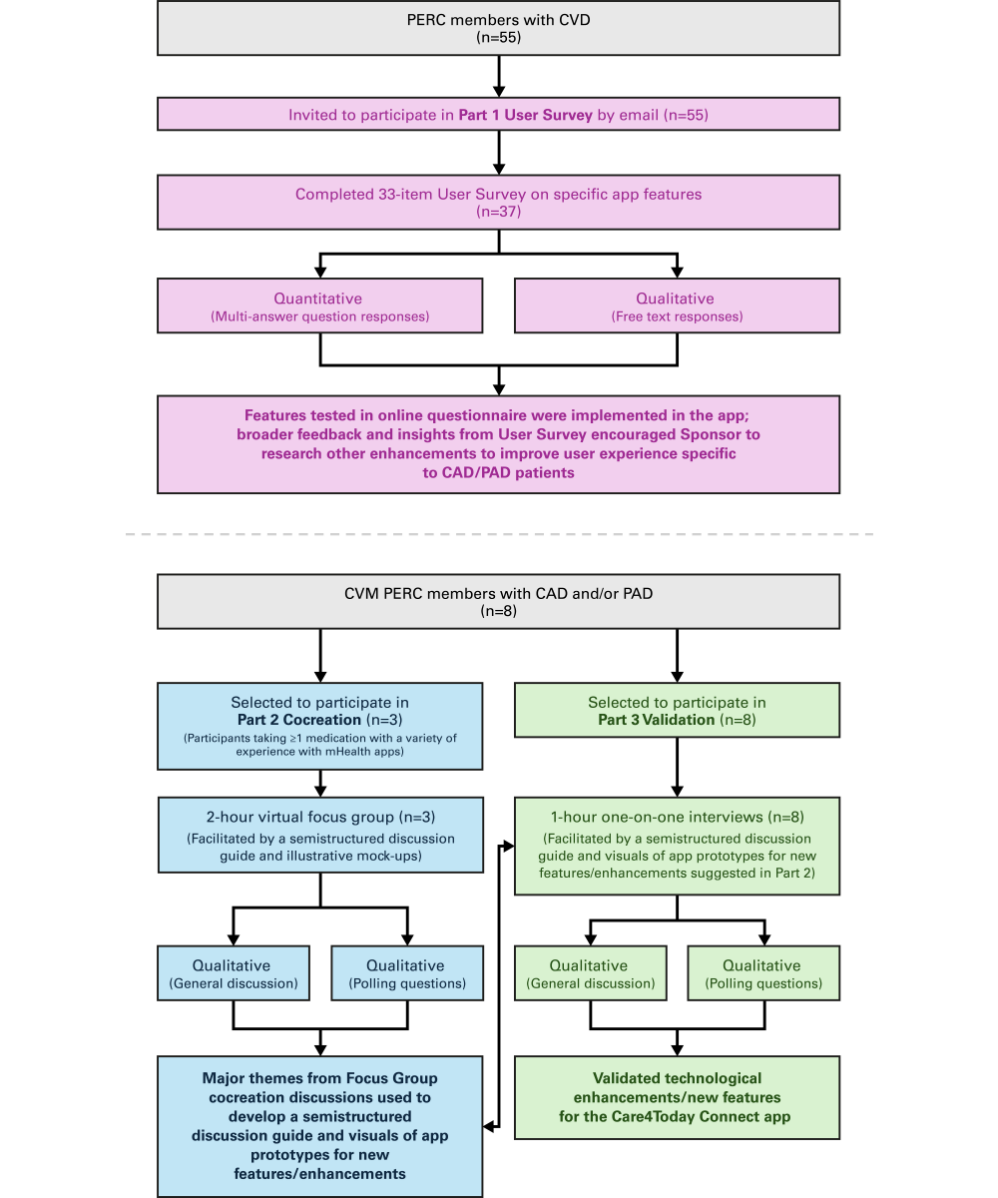
Study design and participant disposition. CAD: coronary artery disease; CVD: cardiovascular disease; CVM: Cardiovascular and Metabolic; mHealth: mobile health; PAD: peripheral artery disease; PERC: Patient Engagement Research Council.

Figure 1 provides a visual diagram of the overall mixed methods design and participant disposition. Participant demographics for each of the 3 parts of the study are described in Table 1. Table 2 describes medication tracking and health experience reporting behavior for participants in parts 2 and 3.

**Table 1:** Participant demographics.

Characteristic			Part 1 (survey; n=37), n (%)		Part 2 (cocreation; n=3), n (%)		Part 3^a^ (validation; n=8), n (%)
**Diagnosis^b^**							
	CAD^c^ and PAD^d^	—^e^		1 (33)		3 (38)	
	PAD alone	Not specified in the responses		2 (67)		5 (63)	
	CAD alone	—		0		0	
**Sex**							
	Male	15 (41)		1 (33)		2 (25)	
	Female	22 (59)		2 (67)		5 (63)	
	Nonbinary	0		—		1 (13)	
**Race**							
	White	10 (27)		1 (33)		4 (50)	
	Black	23 (62)		2 (67)		3 (38)	
	Asian	3 (8)		0		1 (13)	
	Hispanic/Latino or Spanish in origin	3 (8)		0		0	
	American Indian or Alaska Native	1 (3)		0		0	
	Other	1 (3)		—		0	
**Age range (years)**							
	20-29	1 (3)		0		0	
	30-39	1 (3)		0		0	
	40-49	6 (16)		1 (33)		3 (38)	
	50-59	6 (16)		2 (67)		1 (13)	
	60-69	14 (38)		0		3 (38)	
	70-79	9 (24)		0		1 (13)	
**Highest education level**							
	Less than high school	1 (3)		0		0	
	High school	2 (5)		0		1 (13)	
	Some college	4 (11)		1 (33)		1 (13)	
	Trade or technical school	2 (5)		1 (33)		1 (13)	
	Bachelor’s degree	13 (35)		1 (33)		4 (50)	
	Associate degree	1 (3)		0		0	
	Graduate degree	13 (35)		0		1 (13)	
	Other	1 (3)		—		0	
**Region^f,g^**							
	Urban	—		1 (33)		3 (38)^f^	
	Suburban	—		2 (67)		1 (13)	
	Rural	—		0		2 (25)	
**Comorbidities^b,f^**							
	Diabetes	—		2 (67)		5 (63)	
	Any	—		1 (33)		6 (75)	
**Medications/day**							
	<7	—		0		1 (13)	
	7-14	—		2 (67)		5 (63)	
	≥15	—		1 (33)		2 (25)	

^a^3/8 participants from part 3 participated in part 2.

^b^Self-reported diagnosis.

^c^CAD: coronary artery disease.

^d^PAD: peripheral artery disease.

^e^Not applicable.

^f^Data were unavailable for 2 participants in parts 2 and 3.

^g^1 participant responded “urban” but indicated they had previously been “rural.”

**Table 2:** Medication tracking and health experience reporting behavior (parts 2 and 3; n=8).

Participant (parts 2 and 3)	Medication tracking behavior	Health experience reporting behavior
A (parts 2 and 3)	No adherence medication tracking^a^ Uses MyChart for tracking right dosing and frequency for medications Manual pill box used in the morning and afternoon/evening	Uses reminders on continuous glucose monitor and compression boot app devices Uses a journal to record health experiences to be discussed in next health care provider visit
B (parts 2 and 3)	No adherence medication trackinga Uses MyChart for tracking right dosing and frequency for medications Sets up a smartphone alarm twice daily for the morning and afternoon/evening	No health experience tracking or reporting
C (parts 3)	No current medication trackinga Used to track medications on an app	No current health experience tracking or reporting Used to track blood pressure, glucose, bloating, and heart rate on an app, but found it too time-consuming
D (part 3)	No adherence medication trackinga Places pills in a high visibility area	No health experience tracking or reporting
E (part 3)	Uses a weekly pill organizer for drugs for the morning and afternoon/evening	No health experience tracking or reporting
F (part 3)	Uses calendar app, alarms, and reminders to track medication	Keeps track of health experience as part of morning routine Tracks blood pressure, glucose, time in range, weight, and pain on calendar app
G (parts 2 and 3)	Uses retail pharmacy app for tracking medications list	No health experience tracking or reporting
H (part 3)	Uses phone alarms Manual pill box used in the morning and afternoon/evening	Uses health app for tracking glucose (<30 min/d) No other health experience tracking or reporting

^a^Digital or nondigital.

### Part 1: Internet Survey

#### Sample Characteristics

In part 1, a total of 67% (37/55) of participants with cardiovascular disease completed the survey (Table 1). In total, 59% (22/37) participants were female, 59% (22/37) participants were White, 27% (10/37) participants were Black or African American, 62% (23/37) participants were aged ≥60 years, and 81% (30/37) participants had been educated beyond high school. Most (28/37, 76%) had been managing their health condition for >5 years. Overall, 78% (29/37) of survey respondents reported using mHealth apps at least once during the day to help manage their condition, with 35% (13/37) respondents reporting that they used mHealth apps somewhat or very often.

#### Understanding of Existing App Features

When presented with the “Tutorial for New Users” feature (Multimedia Appendix 2), 70% (26/37) of respondents indicated they would continue to use the feature, rather than skip it, and expressed a high level of understanding at each step of the tutorial. Confidence in navigating to various tabs within the Care4Today Connect app was high and most (28/37, 76%) felt at least somewhat likely to use the app after the tutorial.

Respondents understood the concept of the “Earned Points” feature (Multimedia Appendix 2) and most (29/37, 78%) were confident in earning points when using the app but questioned the value of the points reward system. They considered the true value of the app to be in its ability to streamline the functionality of many apps they might be using into one.

Earning points may be motivation for using the app. However, the ability to condense what several apps do into 1 app for me would be a higher motivation. [It] would be nice to focus on that as a convenience and usability feature.

Female participant, 60-69 years, cardiovascular and metabolic disease

#### App Use in Clinical Study

Respondents were asked to assume they had enrolled in a clinical trial that used the Care4Today Connect app and to consider what might drive them to use the app. Motivating factors included contributions to research (33/37, 89%), helping others (29/37, 78%), learning about health/disease (29/37, 78%), improving health (26/37, 70%), better disease management (25/37, 68%), and helping track medications (19/37, 51%). Potential drivers for not using the app included concerns around confidentiality/health data privacy and time obligation.

I would want control of when data is sent to my health care providers and who is authorized to receive that data.

Male participant, 60-69 years, bladder cancer

If it is a huge time obligation, or if it doesn’t sync with my watch, or if it means that I still have to use multiple other apps that I already use on a daily basis...

Female participant, 20-29 years, pulmonary hypertension

Most participants thought the app would be useful in monitoring self-tracked health metrics, such as blood pressure or pain (29/37, 78%), health trends and progress (28/37, 76%), and lifestyle habits (27/37, 73%) (Table 3). Additionally, on a scale of 1 to 10 (where 1 was unlikely and 10 was very likely), most participants (28/37, 76%) selected a response of 7 or higher, indicating that they were likely to recommend the app to a friend or coworker.

**Table 3:** Self-track features of the Care4Today Connect app considered by participants as useful (part 1; n=37). Also, more than 1 item could be selected.

Activity	Respondents, n
Tracking health metrics (eg, blood pressure, pain)	29
Monitoring my health trends and progress	28
Tracking lifestyle habits (daily routine, step count, mood, and sleep)	27
Learning new information about my health	27
Refreshing my knowledge on my health	19
Remembering when my medical appointments are scheduled	18
Remembering to take my medication as prescribed	17
Other	4

### Part 2: Cocreation

#### Sample Characteristics

Three participants from the CAD- or PAD-specific PERC were selected to participate in the virtual focus group in part 2, including 1 male and 2 female patients who were aged between 40 and 59 years, and all of whom were taking 7 or more medications (Table 1).

#### Adding Medication Data for Tracking Features

Currently, adding medication data to the Care4Today Connect app involves manual input of multiple fields to create a customized experience for medication tracking (Multimedia Appendix 3). Illustrative mockups of potential features designed to enable auto-adding medication data to the app were shared with the 3 focus group participants (Multimedia Appendix 4).

Participants saw value in both options to auto-add medication data into the app. Adding medication data via a third-party insurance portal (option 1) was considered the most appealing and convenient solution for the initial setup, allowing a significant number of medications to be added at the same time. Adding medications with OCR technology (option 2) was not considered suitable for initial medication upload due to the associated time burden for patients with CAD, PAD, or both who are typically taking multiple medications; however, this feature was thought to be a better, more intuitive, simpler alternative to option 1 for subsequent additions and changes to medication lists.

For the initial setup, have it imported from your doctor’s office…I would use both but initially I wouldn’t want to take photos of 9 or 10 different bottles to set it up.

Female participant, age 50-59 years, PAD

Other suggestions for simplifying the process of adding medication data included integration with other medical apps, such as MyChart, and pharmacies. Of the 3 focus group participants, 2 already used MyChart for tracking the dosing and frequency of their medications, refills, setting and tracking appointments, contacting their HCPs, and reporting their health experiences (Table 2). Participants also suggested that connection to pharmacies to add medication data may be useful because pharmacy records are typically updated faster than electronic health record data.

#### Self-Reporting Health Experience Features

Participants considered it a simple process to set up the Care4Today Connect app to track and self-report health experiences. While participants were sensitive to the burden of manual reporting of their health experiences, for 67% (2/3) of them this was outweighed by the perceived value of sharing data with their clinical team and having access to a record of their metrics. These 2 participants were willing to manually track their data for approximately 20 minutes/day, a time window corresponding to their disease management routines.

Reminder notifications on apps were considered critical for ongoing tracking, especially when set up to correspond with existing routines. Snooze and follow-up alarm functionalities were requested, rather than a single reminder. Participants expressed a strong preference for wearables or smart devices to overcome the burden of tracking.

Before I track any of these manually, I’d get one of those [smart] watches so it’s tracking for me.

Male participant, age 60-69 years, PAD and CAD

Participants could also see the benefits of immediate feedback based on their self-reported health experiences, such as an automated notification to follow-up with their care team for high blood pressure. A strong preference for HCP-driven notifications and feedback was noted as participants expressed concerns and distrust over generic automated algorithm-generated notifications.

I want the doctors to look at my data and be able to intervene if [that is] something that’s about to happen.

Female participant, age 40-49 years, PAD

No [I would not trust the generic notifications]. That type of stuff will have to come from the doctor. I don’t know who’s behind that information.

Female participant, age 40-49 years, PAD

#### Other Areas for Improvement

Other improvements to the Care4Today Connect app were discussed during the focus group. Areas of concern cited by participants included font sizes, number of fields, and amount of typing required to log into insurance portals to add medication data. One of the recommendations was a multiplatform/multidevice app with the ability to sign into a cloud portal via a laptop or tablet, which would have a larger screen and easier-to-use keyboard than a smartphone. A conversational AI interface was suggested, with language that makes reporting health experiences easier and more natural, intuitive, and engaging (eg, “Are your legs hurting today?” vs “Please report leg pain today”).

We don’t want to load data through the keyboard on [the] phone…most of us are down to 1 or 2 fingers using that. It’s a very slow process as opposed to a regular keyboard even if we’re still just using the same 2 fingers.

Male participant, age 60-69 years, PAD and CAD

### Part 3: Validation

**Figure 2:**
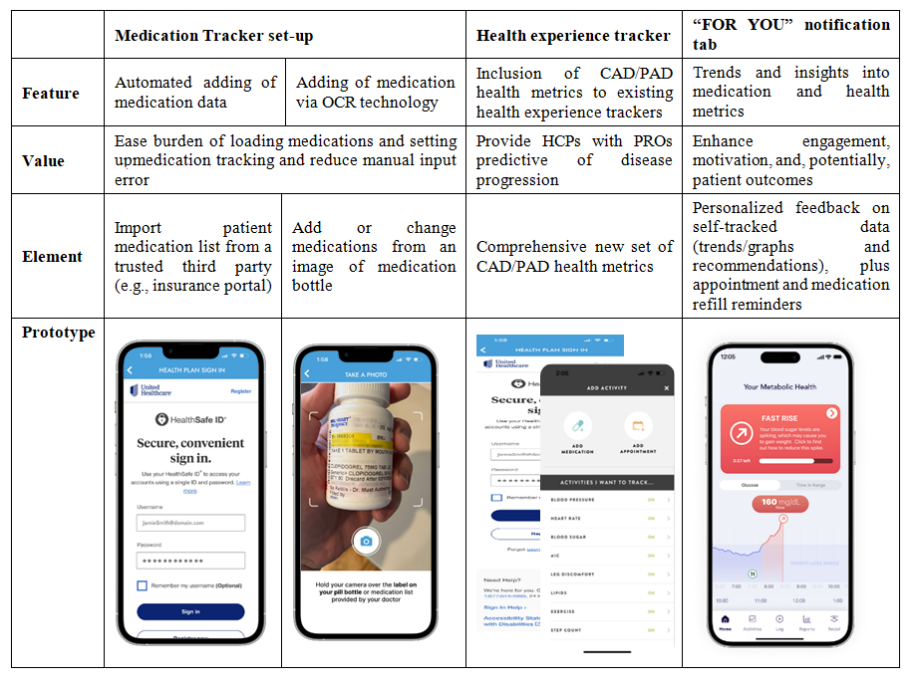
Care4Today Connect app: new features proposed for release 1.0 in CAD or PAD (part 2; n=3). CAD: coronary artery disease; HCP: health care provider; OCR: optical character recognition; PAD: peripheral artery disease; PRO: patient-reported outcome.

In part 3, we sought to validate the additional enhancements proposed during part 2 (Figure 2) through 8 individual interviews with participants with CAD, PAD, or both, using a semistructured discussion guide and visuals of prototypes (Multimedia Appendix 6).

#### Sample Characteristics

Eight of the sponsor’s cardiovascular PERC participated in part 3 of the research. Nearly two-thirds (5/8, 63%) of those interviewed were female, half (4/8, 50%) were White, most (7/8, 88%) had been educated beyond high school, and around two-thirds (5/8, 63%) were aged 50 years and older. Comorbidities were common, with 63% (5/8) reporting comorbid diabetes, and rates of polypharmacy were high, with 88% (7/8) of participants routinely taking ≥7 medications/day. Most participants used an app or a manual pill box as a reminder to take their medication, but only 50% (4/8) of individuals tracked their medications and fewer (3/8, 38%) tracked and reported their health experiences (Table 2).

#### Adding Medication Data Feature

In a study population with marked polypharmacy, participants found value in an app that helps them manage their medications together.

This would be very helpful because it would be all my medications and not just some.

Female participant, age 50-59 years, PAD

**Figure 3:**
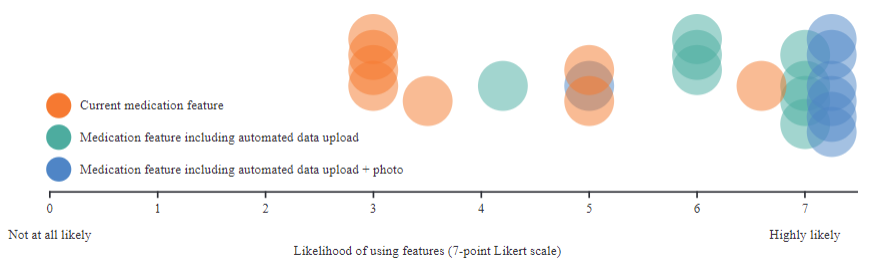
Perceived value of and willingness to use Care4Today medication upload features (part 3; n=8). Each circle represents a participant’s response (3 per participant).

Both proposed medication data features (insurance portal and OCR technology) were well received. When rating the perceived value of each new feature, most participants reacted neutrally to the current manual method of adding medication data for tracking (Figure 3). Most respondents taking ≥7 medications/day (6/7, 85% of participants) considered automatically added medication data from a trusted third party (eg, insurance portal) to be a highly valuable feature. The remaining participant, who was taking 5 medications/day, preferred to upload medications to the app manually rather than via the 2 new features.

While the overall perception of using a third party to add medication data was positive, respondents flagged several potential barriers to its use. Two participants voiced concerns about medication accuracy due to delays in changes to medications on the provider portal. Two participants also worried about the accuracy of medication lists if insurance providers and pharmacies mix claims or if their medication records are not up to date. Another participant mentioned that their small insurer may not be connected to the app.

All respondents saw value in adding medication data via OCR technology for new medications or medication changes. Most found this approach to be preferable to manually typing on their smartphone, particularly due to dexterity issues caused by old age or disease. Only 1 participant felt taking an image of their medication bottle would be difficult, due to shaking hands.

All participants expressed the need to have both options included. Most (6/7, 85%) participants stated that the inclusion of these features increased the likelihood that they would use the Care4Today Connect app.

#### Self-Reporting Health Experiences Feature

Participants reacted positively to a dedicated tool for tracking and sharing their CAD, PAD, or combined health experiences with their HCPs. Most expressed regret about having inaccurate discussions in their HCP visits due to gaps in their self-tracking of health metrics and experiences.

I want to start tracking my symptoms when and where they occur because my doctor does not believe me when I tell him.

Female participant, 40-49, PAD

I often forget what happened last week or last month, so I don’t discuss my old symptoms with my doctor.

Female participant, 60-69, CAD and PAD

Presented with mock-ups of a conversational AI interface for tracking their health experiences (Multimedia Appendix 7), most participants indicated they preferred the more traditional route (ie, inputting data into fields or selecting options). The conversational interface was perceived to be impractical overall, and unsuitable for the reporting of health experience metrics.

I do not want to have a conversation, just log my data.

Male participant, 70-79, CAD and PAD

The text one feels less private.

Female participant, 50-59, PAD

**Figure 4:**
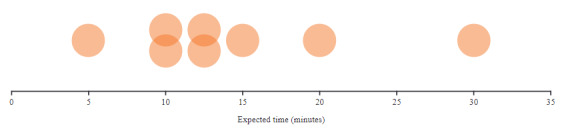
Expected time dedicated to reporting of health metrics and experiences, including symptom and medication reporting (part 3; n=8). Each circle represents 1 participant.

Despite understanding the value of tracking and reporting their data to share with their HCPs, participants were hesitant to dedicate a significant amount of time to this. On average, they were willing to spend 10-15 minutes a day tracking their health metrics and experiences (4 or 5 metrics/day), including medications (Figure 4).

I’d say 10 or 15 minutes. I mean, that’s pretty fair. We waste 10 or 15 minutes a day playing our little online games or something like that, so why not do something that could possibly benefit us, especially if the doctors are directly linked to the app?

Female participant, age 40-49 years, PAD

Certain health data and experiences were more likely to be tracked and reported than others, particularly those recommended by HCPs (Table 4). Participants with existing health or medication routines were most likely to track and report their health experiences.

**Table 4:** Number of participants likely to self-track and report patient-reported outcomes (part 3; n=8).

Health metric	Participants, n
Blood pressure	6
Glucose levels	5
Chest/leg pain or discomfort	5
Swollen feet and limbs, bloating	2
Sleep	2
Weight	2
Heart rate	1
Cramping	1
Shortness of breath	1
Palpitations	1

### “For You” Section

A “For You” section in the app was considered essential to create an all-encompassing platform for managing CAD, PAD, or both. Personalized notifications were believed to be of value if they were validated by an HCP, rather than being an automated response from the app. These might include recommendations or actions for a particular health metric (eg, go for a walk), alerts to contact the care team (eg, call the nurse or schedule an appointment), and changes in medication.

I like it because you’re directly communicating with your doctor instead of waiting a month in pain.

Female participant, age 40-49 years, PAD

I think it [rule-based notifications] would still be appreciated, but I think it would be deeply appreciated coming from the provider’s practice.

Female participant, age 60-69 years, PAD and CAD

### Other Areas for Improvement

The user interface of the Care4Today Connect app was well received, particularly because of its simplicity, design, and intuitive workflow. There was an agreement with earlier feedback from the part 2 virtual focus group that the visual design could be improved by increasing button size, font size, and font contrast, and altering colors, to address accessibility and visibility challenges.

## Discussion

### Principal Results

This mixed methods research identified technological app enhancements to the Care4Today Connect, including improving the utility of the medication tracking as well as improving self-reported health data and experiences with relevant care team feedback, to optimize its ability to meet the specific requirements of patients with CAD, PAD, or both.

The existing Care4Today digital platform is continuously updated to enhance its utility. An initial internet survey of a broad group of cardiovascular participants, including those with CAD and PAD indicated a general understanding of key features, as well as opportunities for further enhancements. Based on this, we asked participants with CAD, PAD, or both for suggestions on improving the app. In a virtual focus group and individual interviews, participants told us they could see value in using technology to help add their medication data for tracking because it could reduce the user burden of having to manually add medication data. Participants also indicated that they would find self-reporting their health experiences valuable if the time obligation was not onerous. Further, respondents were interested in personalized in-app feedback from their care team based on their self-reported medication tracking and health experiences.

### Comparison With Prior Work

#### Adding Medication Data Feature

Polypharmacy is common in the CAD, PAD, or both populations, who typically comprise an older cohort with multiple comorbidities. In our focus group sample, 7/8 participants reported taking ≥7 medications/day, with 2 participants taking >15 medications/day. This is consistent with data from a claims-based study (n=148), in which 91% of patients with CAD were found to be taking ≥5 medications, with 74% taking ≥5 cardiovascular medications [10].

Polypharmacy is linked to both medication errors [11] and nonadherence [12]. mHealth apps provide a patient-centered means of targeting medication adherence [22]. Participants in our study stated that they would welcome multiple features on the Care4Today Connect app to allow for automated medication data to assist with medication tracking. Minimizing the reliance on manual input of data, by offering automated options, should reduce both the time burden associated with manual input and the potential for data entry errors. While older adults with CAD are proficient users of mobile apps and find them useful for medication adherence [23], our research highlights visibility and dexterity challenges as barrier to their use, particularly on a small screen. Automatic addition of medications using OCR technology has been shown to track medication adherence accurately [24], and optimization and flexibility of medication data input are commonly requested by users of medication adherence apps [22].

#### Self-Reporting Health Experiences Feature

A recent poll suggests that 2 in 5 US adults use mHealth apps, with at least half of them using the technology daily [25]. There is clear familiarity with this kind of technology among the general population and evidence of improvements in adherence and short- and long-term outcomes in people with CAD, PAD, or both who use mHealth apps [14-17]. Nevertheless, many of those in our study were either not currently tracking their medication and health experiences or were tracking these metrics through different channels or methods, such as pill boxes. In total, 78% of participants in part 1 said they used mHealth apps to help manage their disease, but only half the patients with CAD, PAD, or both in parts 2 and 3 reported routinely tracking their medications, with even fewer tracking and reporting their health experiences. Time constraints were identified as a barrier.

The ability to connect with their HCP or clinical team was positively received and participants were interested in additional notifications if they came with a personalized recommendation from their HCP. Immediate feedback on health metrics can enhance user engagement, motivation, and, potentially, patient outcomes by providing the user with a sense of progress. Indeed, a questionnaire-based survey of 180 patients with PAD concluded that information, monitoring, and feedback were the most relevant mHealth app components for this population [26].

### Strengths and Limitations

Patient feedback is essential for the optimization of the content and quality of digital health tools. The cocreation and validation approach used in our research ensured that participants with CAD, PAD, or both were involved in the co-design and refinement of potential enhancements to the Care4Today digital platform that would address their unique needs. Both quantitative and qualitative components ensured that valuable patient insights and rich context around their choices were captured to guide future app development. However, this was exploratory research and, as such, had several limitations. First, as with many studies of this nature, our focus group and interviews involved only a small number of participants with CAD or PAD, or both. Hence, our findings are not generalizable to t the broader population with CAD, PAD, or both. Second, while the study sample was ethnically and demographically diverse, participants had been invited to participate from existing PERC programs and, as such, self-selection bias resulted in a sample of participants that were more engaged and aware of their disease than the wider population of those with CAD or PAD, or both. This could potentially influence responses toward greater familiarity with mHealth apps. Third, CAD and PAD diagnoses were self-reported. There is a risk that self-reported diagnoses may differ from clinical diagnoses depending on the quality of patient–clinician communication, time since diagnosis, and the health literacy of the patient. Finally, employees of the Sponsor were present during virtual sessions. However, CorEvitas and ZS Associate researchers introduced themselves including first name, company affiliation, and research objectives. The facilitator’s introduction informed participants of sponsor’s presence but also included instruction that the aim was to gather participants’ honest feedback and there were no wrong answers.

### Future Directions

Patients and HCPs are key stakeholders of any digital health tool, including the Care4Today Platform. Feedback from both groups is inherent to the digital platform’s usability and adaptability across the health care system. While this article has focused on the patient, the Care4Today team has also engaged key opinion leaders in the Cardiovascular space, which include HCPs and professional organizations. There is an opportunity to take learnings from both engagements and explore a study where codevelopment and validation are conducted with both patients and HCPs.

### Conclusions

The Care4Today digital platform is focused on improving medication adherence, enabling self-reporting of health experiences providing patient education, enhancing connection with HCPs, and facilitating data and analytics learning across select disease areas. Our exploratory mixed methods research sought to identify how to improve the overall experience of patients with CAD or PAD, or both using Care4Today Connect. The goal was to understand patient insights and preferences on how they could add and self-report medication and health experience data. Key takeaways include recommendations to focus on enhancements that could reduce user burden through automation and technology, and foster HCP connection with personalized feedback. Incorporating new features that have been ideated and validated by patients, who are also end users, is crucial to the development and utility of digital apps. Through this research, the Care4Today team can prioritize the next iteration of the platform to optimize the experience for both patients and health care teams.

## Appendix

Multimedia Appendix 1 Janssen’s Cardiovascular Metabolic Patient Engagement Research Council: eligibility criteria.

Multimedia Appendix 2 Part 1 internet survey.

Multimedia Appendix 3 Screenshots of the existing feature for adding medication data manually on the Care4Today Connect app.

Multimedia Appendix 4 Illustrative mock-ups presented to facilitate discussion during the part 2 virtual focus group: (A) features for adding medication data and (B) features for health experience reporting.

Multimedia Appendix 5 Prototypes for the Care4Today Connect app presented during the part 3 interviews: (A) automatic adding of medication data concept testing and (B) tracking and sharing of health experiences value proposition testing.

Multimedia Appendix 6 Semistructured discussion agenda for individual validation interviews (part 3).

Multimedia Appendix 7 Example of a conversational user interface on a mobile health app.

## Abbreviations

CAD: coronary artery disease
HCP: health care provider
HIPAA: Health Insurance Portability and Accountability Act
IRB: institutional review board
mHealth: mobile health
OCR: optical character recognition
PAD: peripheral artery disease
PERC: Patient Engagement Research Council

## Data Availability

The data that support the findings of this study are available on request from the corresponding author. The data are not publicly available due to privacy or ethical restrictions.
