# Supplementary material for: Optimization of the Care4Today Digital Health Platform to Enhance Self-Reporting of Medication Adherence and Health Experiences in Patients With Coronary or Peripheral Artery Disease: Mixed Methods Study
Source: JMIR Cardio. 2025 Mar 17;9:e56053. doi: 10.2196/56053 (PMC11959196; doi:10.2196/56053)
Supplement: Multimedia Appendix 1 [file cardio_v9i1e56053_app1.pdf]

MULTIMEDIA APPENDIX

Multimedia Appendix 1. Janssen’s Cardiovascular Metabolic Patient Engagement Research Council: Eligibility Criteria.

	**Inclusion criteria**	**Exclusion criteria**
**PAD**	▪ Diagnosis of PAD via ABI testing<br>▪ Acute (intervention within past 6 months) or chronic	**CAD/PAD**<br>▪ Diagnosed with unrelated bleeding disorder<br>▪ MI >12 months[a]<br>▪ Diagnosis other than CAD/PAD requiring AC (ie, NVAF)
**Polyvascular population**	▪ Diagnosis of CAD via cardiac catheterization by a cardiologist<br>▪ CAD with history of MI >12 months[a]<br>▪ With or without diabetes	
**VTE**	▪ Diagnosis of PE/DVT or DVT only<br>▪ Diagnosis within 6 months on active treatment<br>▪ Diagnosed with ≥2 VTE events >12 months ago	▪ Aged <40 years<br>▪ Diagnosed with unrelated bleeding disorder<br>▪ Diagnosis other than VTE requiring AC (ie, NVAF)
**Cardiovascular/ atrial fibrillation**	▪ Aged ≥65 years<br>▪ With or without atrial fibrillation	

[a]If accompanied by PAD of lower extremities.

ABI: ankle brachial index; AC: anticoagulant; CAD: coronary artery disease; DVT: deep vein thrombosis; MI: myocardial infarction; NVAF: nonvalvular atrial fibrillation; PAD: peripheral artery disease; PE: pulmonary embolism; VTE: venous thromboembolism.
