# Supplementary material for: Optimization of the Care4Today Digital Health Platform to Enhance Self-Reporting of Medication Adherence and Health Experiences in Patients With Coronary or Peripheral Artery Disease: Mixed Methods Study
Source: JMIR Cardio. 2025 Mar 17;9:e56053. doi: 10.2196/56053 (PMC11959196; doi:10.2196/56053)
Supplement: Multimedia Appendix 2 [file cardio_v9i1e56053_app2.pdf]

Multimedia Appendix 2. Part 1 Internet Survey

Upfront Questions

1. Which Janssen PERC do you participate in? [Select all that apply]
   - Ankylosing spondylitis
   - Bladder cancer
   - Cardiovascular
   - Inflammatory bowel disease
   - Multiple myeloma
   - Multiple sclerosis
   - Pulmonary hypertension
   - Prostate cancer
   - Psoriatic arthritis
   - Psoriasis
   - Stem cell
   - I’m not sure

2. How long have you been managing your health condition(s)? [Select one answer]
   - Less than 1 year
   - 1–2 years
   - 2–5 years
   - 5–10 years
   - More than 10 years

3. How often do you use health apps (an app designed to help you manage your health/disease condition) during the day? [Select one answer]
   - Very often
   - Somewhat often
   - Sometimes
   - Rarely
   - Not at all

Tutorial for New Users

4. At this point, would you feel compelled to continue through the tutorial or skip the tutorial? [Select one answer]
    - Continue through the tutorial
    - Skip the tutorial
    - Skip the tutorial and return to it later

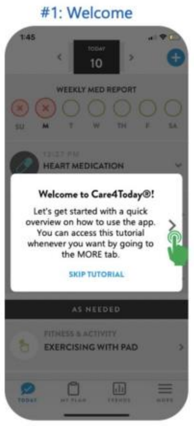

5. On a scale of 1 to 5 (where 1 is strongly disagree and 5 strongly agree), please indicate whether you agree or disagree with each statement.
    - I understand how to add activities to the My Plan tab
    - I understand that I must click the plus sign in the upper right-hand corner to add reminders
    - I understand that I can add different activities such as medication reminders and appointments to the app

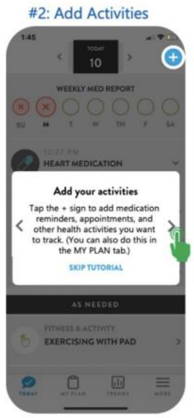

6. Do you have any questions about this screen? **Please do not mention specific medications.** [Free text response]

7. On a scale of 1 to 5, (where 1 is strongly disagree and 5 strongly agree), please indicate whether you agree or disagree with each statement.
    - I understand how to navigate the Today tab
    - I understand that I can track activities and read educational articles within the app

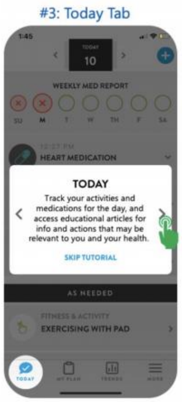

8. Do you have any questions about this screen? **Please do not mention specific medications.** [Free text response]

#4: My Plan Tab

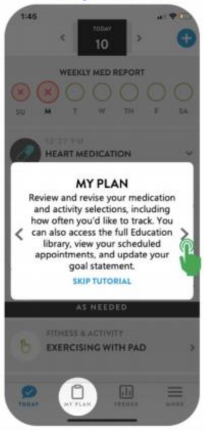

9. On a scale of 1 to 5, (where 1 is strongly disagree and 5 strongly agree), please indicate whether you agree or disagree with each statement.

- I understand how to navigate the “My Plan” section
- I understand what actions I can take in the “My Plan” section

10. Do you have any questions about this screen? **Please do not mention specific medications.** [Free text response]

#5: My Trends Tab

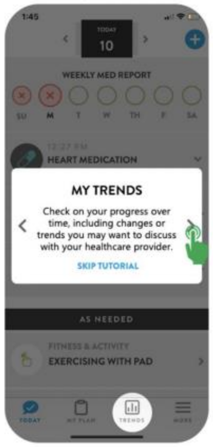

11. On a scale of 1 to 5, (where 1 is strongly disagree and 5 strongly agree), please indicate whether you agree or disagree with each statement.

- I understand how to navigate the “My Trends” section
- I understand that I can review my progress in the “My Trends” section

#6: More Tab

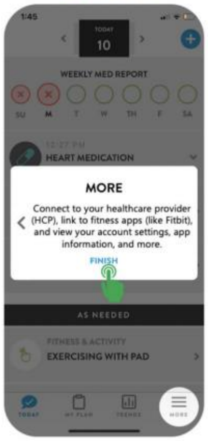

12. Do you have any questions about this screen? **Please do not mention specific medications.** [Free text response]

13. On a scale of 1 to 5, (where 1 is strongly disagree and 5 strongly agree), please indicate whether you agree or disagree with each statement.

- I understand how to navigate the “More” section
- I understand what actions I can take in the “More” section

#3: Today Tab

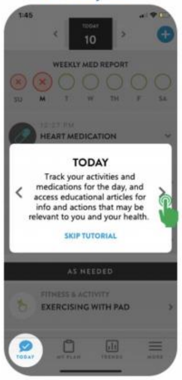

14. Do you have any questions about this screen? **Please do not mention specific medications.** [Free text response]

15. On a scale of 1-5 (where 1 is not at all confident and 5 is very confident, how confident are you that you can navigate the **Today** tab? (not at all confident, not very confident, neutral, somewhat confident, very confident)

16. How confident are you that you can navigate the **My Plan** tab?

17. How confident are you that you can navigate the **My Trends** tab?

18. How confident are you that you can navigate the **More** tab?

19. How confident would you feel using the Care4Today® app after viewing this tutorial?

20. How likely would you be to use the Care4Today® app after viewing this tutorial? (not at all likely, not very likely, neutral, somewhat likely, very likely)

21. If you could change anything about the content in the tutorial feature, what would it be? [Free text response]

Earned Points Feature

22. On a scale of 1 to 5, (where 1 is strongly disagree and 5 strongly agree), please indicate whether you agree or disagree with each statement

- I understand how to mark an article as read in the app
- I understand how to select an article in the app
- I understand that I will receive points for completing certain activities in the app

23. Do you have any questions about these screens? **Please do not mention specific medications.** [Free text response]

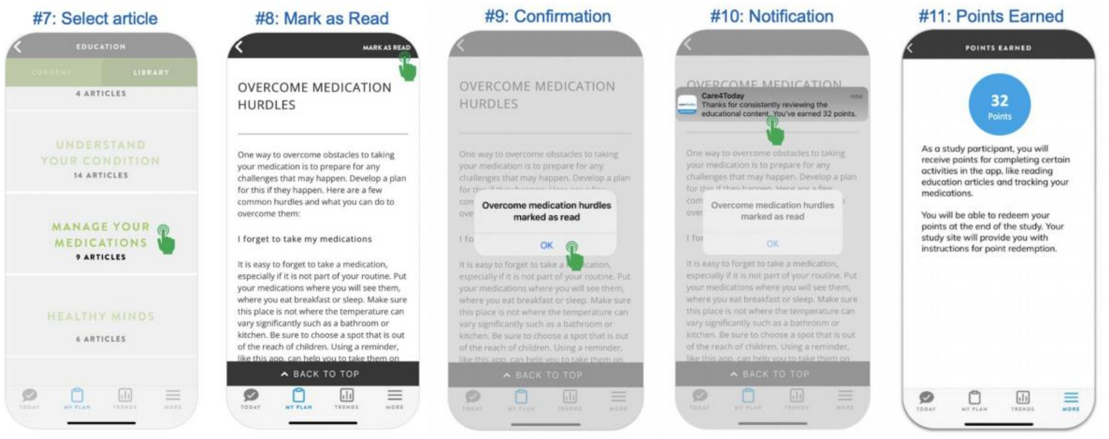

24. On a scale of 1 to 5, (where 1 is strongly disagree and 5 strongly agree), please indicate whether you agree or disagree with each statement.

- I'm interested in earning points through the app to show my progress
- I would use this app because of the earned points feature
- I am confident I can earn points in the app

25. What would you change about the Earned Points feature, if anything? **Please do not mention specific medications.** [Free text response]

App in Clinical Study

26. Assume you have enrolled in a clinical trial that uses the Care4Today® app. What reasons might motivate you to use the app for this study? [Select all that apply]

- To contribute to research
- To help others
- To learn more about my health/disease condition
- To improve my health
- To better manage my disease conditions
- To help me track my medications
- Other. Please do not mention specific medications. [Free text response]

27. What reasons might make you not want to use the app for this study? [Free text response]

28. How often would you be willing to use this app for a study? [Select one answer]
- Less than once a week
- Once per week
- A few times per week
- Once per day
- Multiple times a day

29. How long would you be willing to use a health app in a study setting? [Select one answer]
- Less than 3 months
- 4-6 months
- 7-9 months
- 10-12 months
- More than 12 months

30. With which of the following items do you believe the Care4Today® Connect app would help you? [Select all that apply]
- Tracking health metrics (blood pressure, pain, etc)
- Monitoring my health trends and progress
- Tracking lifestyle habits (daily routine, step count, mood, sleep…)
- Learning new information about my health
- Refreshing my knowledge on my health
- Remembering when my medical appointments are scheduled
- Remembering to take my medication as prescribed
- Other [free text response]

31. The team who developed the Care4Today® Connect app is currently developing a website and community/social media page. What type of information would you find valuable on these platforms? **Please do not mention specific medications.** [Free text response]

32. Based on the features you reviewed today, on a scale of 1 to 10 (where 1 is unlikely and 10 is very likely), how likely are you to recommend the Care4Today® Connect app to a friend or coworker?

33. Please explain why you chose your answer. **Please do not mention specific medications.** [Free text response]
