# Supplementary material for: Optimization of the Care4Today Digital Health Platform to Enhance Self-Reporting of Medication Adherence and Health Experiences in Patients With Coronary or Peripheral Artery Disease: Mixed Methods Study
Source: JMIR Cardio. 2025 Mar 17;9:e56053. doi: 10.2196/56053 (PMC11959196; doi:10.2196/56053)

**Multimedia Appendix 3.** Screenshots of the existing feature for adding medication data manually on the Care4Today® Connect app.

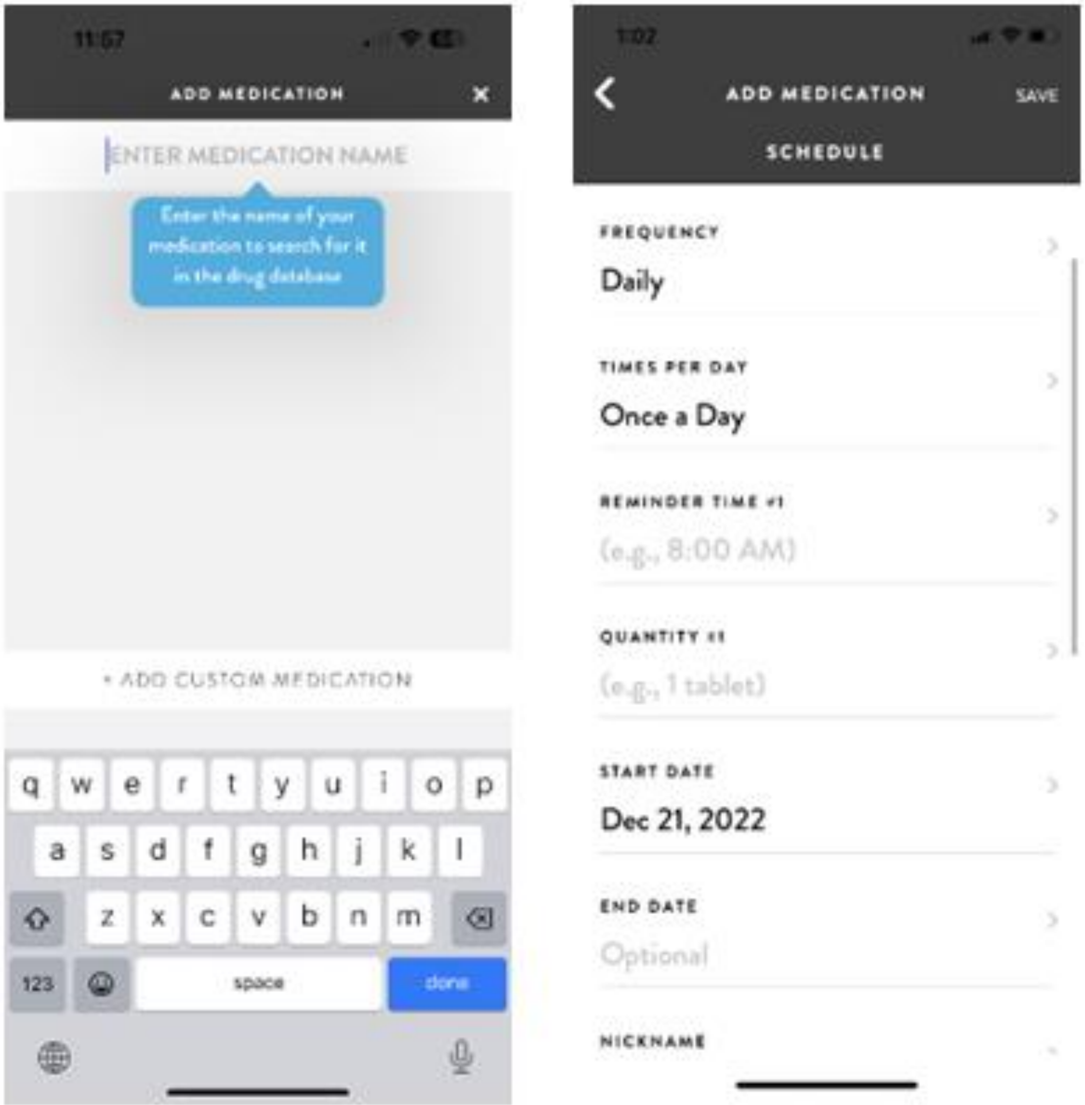

Supplement: Multimedia Appendix 3 [file cardio_v9i1e56053_app3.pdf]
