# Supplementary material for: Optimization of the Care4Today Digital Health Platform to Enhance Self-Reporting of Medication Adherence and Health Experiences in Patients With Coronary or Peripheral Artery Disease: Mixed Methods Study
Source: JMIR Cardio. 2025 Mar 17;9:e56053. doi: 10.2196/56053 (PMC11959196; doi:10.2196/56053)

Multimedia Appendix 4. Illustrative mock-ups presented to facilitate discussion during the Part 2 virtual focus group. (A) Features for adding medication data; (B) Features for health experience reporting.

(A) Features for adding medication data

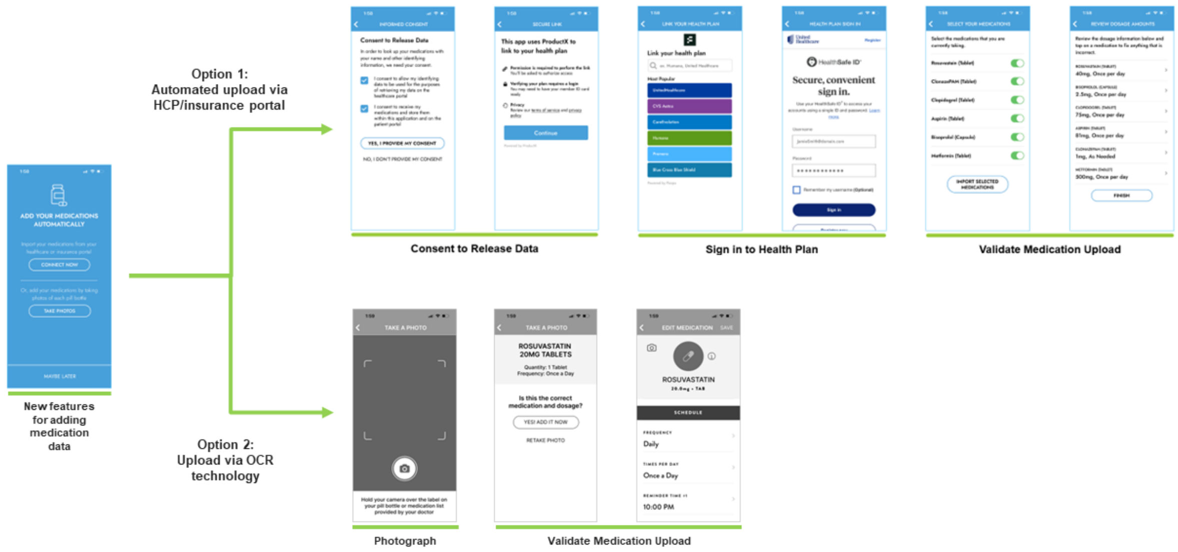

(B) Features for health experience reporting

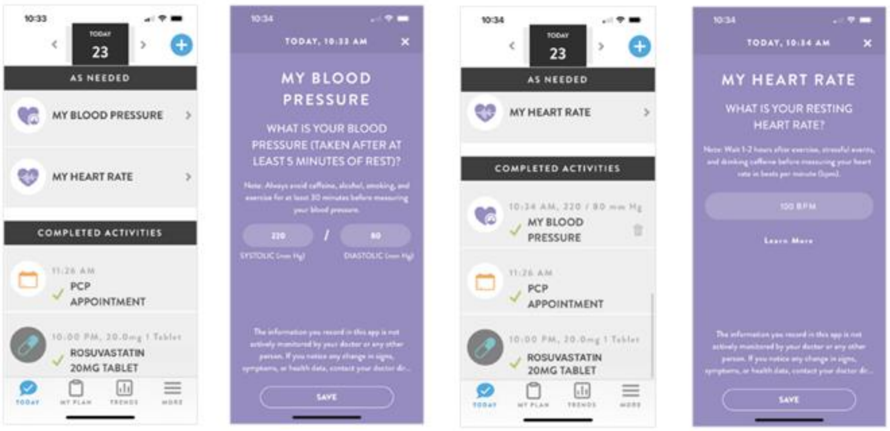

Supplement: Multimedia Appendix 4 [file cardio_v9i1e56053_app4.pdf]
