# Supplementary material for: Optimization of the Care4Today Digital Health Platform to Enhance Self-Reporting of Medication Adherence and Health Experiences in Patients With Coronary or Peripheral Artery Disease: Mixed Methods Study
Source: JMIR Cardio. 2025 Mar 17;9:e56053. doi: 10.2196/56053 (PMC11959196; doi:10.2196/56053)

Multimedia Appendix 5. Prototypes for the Care4Today® Connect app presented during the Part 3 interviews. (A) Automatic adding of medication data concept testing; (B) tracking and sharing of health experiences value proposition testing.

(A) Automatic adding of medication data concept testing

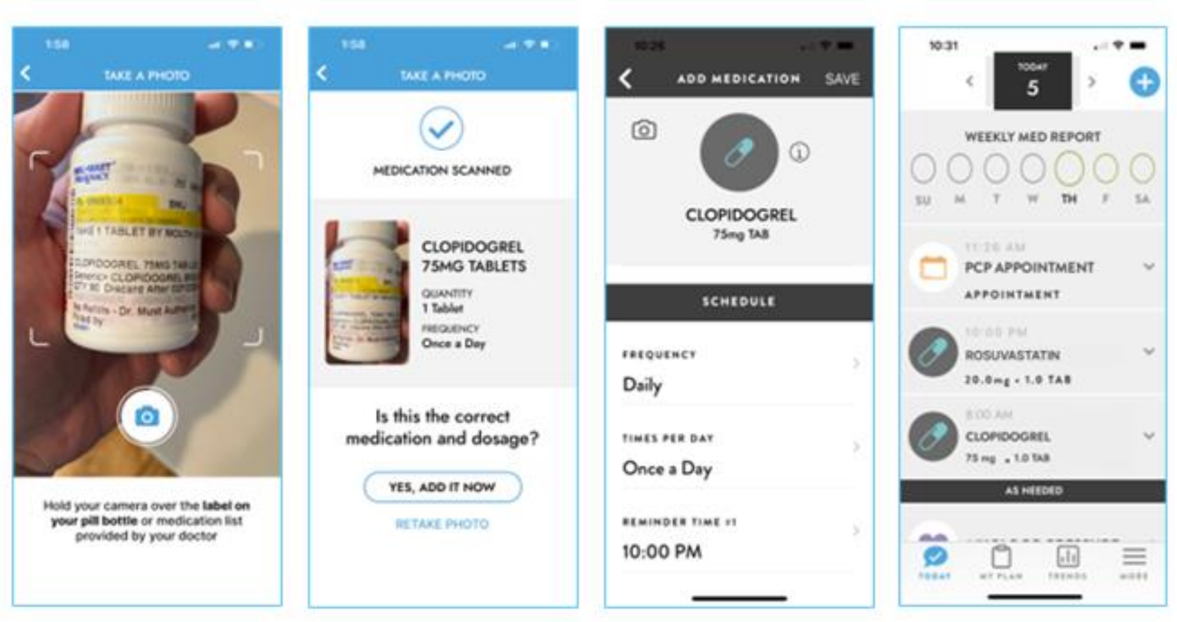

(B) Tracking and sharing of health experiences value proposition testing

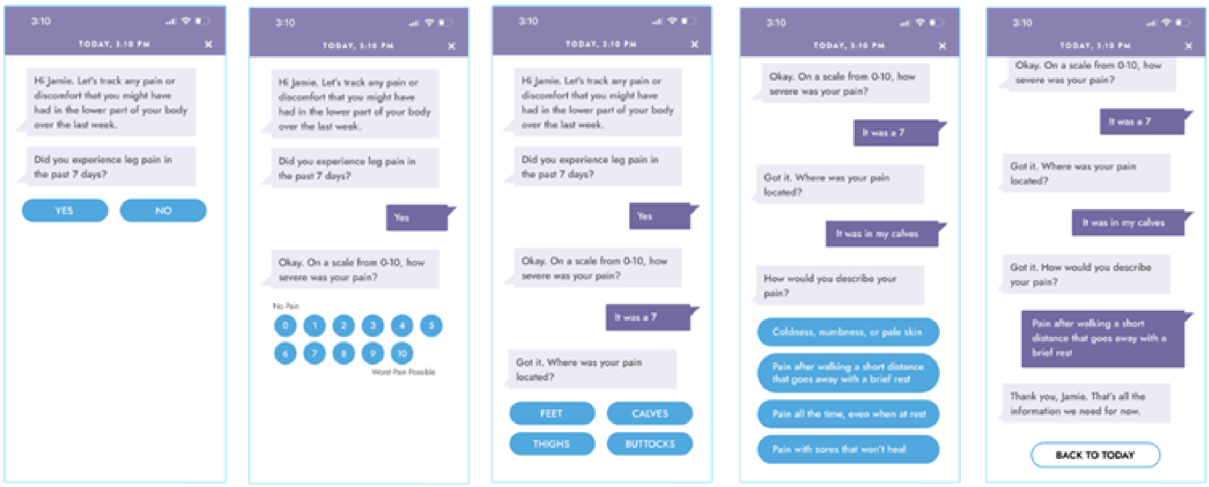

Supplement: Multimedia Appendix 5 [file cardio_v9i1e56053_app5.pdf]
