# Supplementary material for: Optimization of the Care4Today Digital Health Platform to Enhance Self-Reporting of Medication Adherence and Health Experiences in Patients With Coronary or Peripheral Artery Disease: Mixed Methods Study
Source: JMIR Cardio. 2025 Mar 17;9:e56053. doi: 10.2196/56053 (PMC11959196; doi:10.2196/56053)
Supplement: Multimedia Appendix 6 [file cardio_v9i1e56053_app6.pdf]

Multimedia Appendix 6. Semi-structured discussion agenda for individual validation interviews (Part 3).

Section	Objectives	Duration
**Introduction and disclosures**	▪ Introduce moderator and disclaimers<br>▪ Introduce topic of the discussion<br>▪ Gather high-level respondent background	5 min
**Current experience and unmet need validation**	▪ Validate pain points around medication data upload and tracking	10 min
**Medication automated upload concept and value proposition testing**	▪ Assess resonance of the feature's value proposition and impact of new functionality on unmet need alleviation<br>▪ Gather feedback on concept and identify new factors that can improve willingness to use, reduce friction of new tasks, and encourage behaviors (e.g. incentives, instructions, workflow, desired user experience)	20 min
**Tracking and sharing of data value proposition testing**	▪ Assess resonance of value proposition to track data for HCPs to remotely monitor patient<br>▪ Assess willingness to track data<br>▪ Assess willingness to sharing their health data<br>▪ Gather feedback on, and identify new factors that can improve willingness to use, reduce friction of new tasks, and encourage behaviors (e.g. incentives, instructions, workflow, desired user experience)	20 min
**Wrap up**	▪ Closing thoughts<br>▪ Wrap up and close	5 min

HCP: healthcare provider.
